# The Role of Caregivers in Preventing and Managing Malnutrition Among Older Adults: A Narrative Review

**DOI:** 10.3390/nu18060982

**Published:** 2026-03-19

**Authors:** Michela Zanetti, Paolo De Colle, Matteo Bianchini, Dario Calandrino, Sabrina Rampazzo, Luisa Solimando, Nicola Veronese

**Affiliations:** 1 Postgraduate Residency Program in Geriatrics, Department of Medical, Surgical and Health Sciences, University of Trieste, 34127 Trieste, Italy; matteo.bianchini@studenti.units.it; 2 Geriatric Clinic, Maggiore University Hospital, Piazza Dell’ospitale 1, 34127 Trieste, Italy; paolo.decolle@asugi.sanita.fvg.it (P.D.C.); dario.calandrino@asugi.sanita.fvg.it (D.C.); 3 Undergraduate Program in Dietetics, Department of Medical, Surgical and Health Sciences, University of Trieste, 33170 Pordenone, Italy; sabrina.rampazzo@asfo.sanita.fvg.it; 4 Faculty of Medicine, Saint Camillus International University of Health Sciences, 00131 Rome, Italy; luisa.solimando@unicamillus.org (L.S.); nicola.veronese@unicamillus.org (N.V.); 5 Primary Care Department, Unità Locale Socio-Sanitaria 3 “Serenissima”, 30174 Venice, Italy

**Keywords:** older adults, caregiver, malnutrition, nutritional risk, aging, nutrition intervention

## Abstract

**Background/Objectives**: Approximately 1 in 10 community-dwelling older adults are affected by or at risk of malnutrition, and this prevalence increases to nearly 1 in 3 among those receiving home care or recently hospitalized, contributing to higher rates of frailty, falls, hospitalization, functional decline, and mortality. Many of these individuals depend on informal or family caregivers for nutritional care, including assistance with grocery shopping meal preparation, feeding, and monitoring dietary intake. Thus, informal caregivers play an increasingly central role in supporting dietary intake and maintaining nutritional status. This narrative review aims at assessing the relationship between informal caregiver involvement and malnutrition in community-dwelling older adults who are dependent for nutritional-related needs, summarizing evidence on caregiver’s role and caregiver-associated determinants of malnutrition, as well as on interventions that incorporate caregivers into nutrition care. We discuss factors associated with malnutrition in later life, with particular emphasis on caregiver knowledge, burden, interventions and outcomes. In addition, caregiver-inclusive models of care and tools, including nutrition education and guidelines/recommendations, medical nutrition therapy, and multidisciplinary care models will be addressed. **Methods**: A structured review of the literature was conducted (date of last search December 2025), searching multiple databases for pertinent articles. Following identification of eligible articles for inclusion, a narrative synthesis of evidence was completed. **Results and Conclusions**: Despite the high degree of heterogeneity in methodology, observational studies demonstrate that several caregiver attributes influence the nutritional status of care recipients, including caregiver’s own nutritional status, burden, knowledge and literacy, psychosocial, environmental and economic factors. Intervention studies show that caregiver-focused, -inclusive and -delivered interventions have a positive impact on several outcomes, including improved older care recipient dietary intakes, nutritional status and quality of life without impacting on caregiver burden. Thus, strengthening caregiver support and integrating caregivers into nutrition screening and intervention frameworks may represent a critical opportunity to reduce malnutrition risk and improve health outcomes among older adults. Still, significant gaps remain in caregiver-focused intervention research, particularly in diverse cultural and social contexts.

## 1. Introduction

With the progressive aging of the population, a growing proportion of older adults are at risk of developing malnutrition or are already malnourished. The prevalence of malnutrition varies by setting (lower in the community versus institutions and hospitals), geographical area and demographics, being highest in oldest-old individuals [1,2]. However, since the majority of older adults live in the community, the largest number of malnourished individuals is found in this setting [3]. Malnutrition exists in different forms, including undernutrition, micronutrient deficiencies, disease-related malnutrition overweight, and obesity [4]. However, in community-dwelling older adults, the most commonly represented forms of malnutrition are protein–energy malnutrition (PEM), a subtype of undernutrition resulting from inadequate intake of protein and calories and micronutrient deficiencies [5,6]. PEM and micronutrient deficiencies are often under-recognized and have been associated with serious consequences including impaired physical, cognitive and functional status, poor quality of life, weakened immune response, increased risk of osteoporosis and fractures and increased morbidity and mortality [1,5,6,7]. Since malnutrition is a modifiable risk factor for adverse health and functional outcomes, it is important to promptly recognize its signs and symptoms in order to ensure adequate interventions. Older adults in both the community and in long-term care are increasingly dependent on formal and informal caregivers for several activities [8]. They include nutrition-related needs, like food provision, meal preparation and assistance, hydration monitoring [9] and managing common age-related symptoms with nutritional impact, such as poor appetite, changes in taste and smell and problems with swallowing, chewing and missing teeth and poorly fitting prostheses [10]. Polypharmacy typical of frail older adults increases the risk of malnutrition and nutrient interactions, as medications can cause anorexia, interfere with nutrient absorption and metabolism and induce specific nutrient deficiencies [11,12]. This growing dependence highlights the crucial role played by caregivers with specific responsibilities ranging from occasional help to managing complex health situations [13]. Despite these responsibilities, caregivers often have limited knowledge about malnutrition and its health consequences as well as about optimal dietary patterns, energy and protein requirements and general diet-related needs, substitutions, and modifications for their care recipients [14,15]. With the increasing need for care and recipients’ complexity, particularly in older adults with specific medical conditions with a high nutritional impact (such as dementia, stroke or chronic renal failure), the caregiver’s role becomes more complicated and stressful [16]. Thus, it is very relevant to focus on caregiver support, training and involvement in nutritional care teams to prevent malnutrition in older adults with limited independence. In addition, potential barriers should be identified and addressed. Despite the relevance of the caregiver–older care recipient dyad on the management of malnutrition in the community, the literature remains fragmented regarding how informal caregivers recognize, prevent, and manage malnutrition in the home setting, with limited synthesis of their roles, challenges, and support needs; therefore, this narrative review aims to critically summarize and integrate existing evidence to clarify current knowledge gaps and inform future research and practice.

To comprehensively address the topic of this narrative review, we formulated and examined the following key questions:

(1) What is the burden of malnutrition among community-dwelling older adults, both in general and specifically among those cared for by informal caregivers?
(2) Which are the caregiver attributes that contribute to malnutrition in older care recipients?
(3) What evidence is available for nutritional interventions targeting informal caregivers of older adults and their outcomes?
(4) What are the barriers and facilitators to implementing caregiver-driven nutritional interventions, as well as their implications for policies and practices?

## 2. Materials and Methods

For this review a formal literature search was the primary method of identifying relevant manuscripts. Given the high prevalence of malnutrition among community-dwelling older adults and its burden on those who are nutritionally dependent, we focused on this population. Nutrition dependence as a result of IADL or ADL impairment, cognitive impairment or physical limitations was defined as the need for assistance with food access (e.g., shopping or procurement), meal preparation, feeding assistance, and/or intake monitoring. Since nutritionally dependent community-dwelling older adults frequently rely on informal caregivers for day-to-day nutritional care, this narrative review specifically focuses on this type of caregivers to better understand their role, challenges, and impact on nutritional outcomes in care recipients. Formal caregivers were excluded because their responsibilities are typically structured, time-limited, and embedded within professional care systems, which differ substantially from the continuous non-structured support provided by informal caregivers. Although this study was conducted as a narrative review, the study selection process followed structured search and screening procedures to enhance transparency and reproducibility. First the electronic databases PubMed, Scopus and Web of Science were searched to identify articles of interest from inception to December 2025 (date of last search) with the initial search limited to the title and abstract. The search strategy combined terms related to:

(1) Malnutrition (e.g., malnutrition, undernutrition, nutritional deficiency, protein-energy malnutrition, nutrition risk);
(2) Older adults (e.g., older adults, elderly, geriatric, aged);
(3) Community setting (e.g., community-dwelling, community-living, home-dwelling, non-institutionalized);
(4) Informal caregivers (e.g., caregiver, family caregiver, informal caregiver, spousal caregiver);
(5) Caregivers’ delivered nutritional interventions.

Specific strings used in the literature search are shown in Supplementary Table S1.

Reference lists of included articles and relevant reviews were manually screened to identify additional pertinent studies. Care was taken to avoid double counting of data when both reviews and their included primary studies were cited. Studies were considerable eligible if they:

(1) Included community-dwelling adults aged ≥65 years;
(2) Examined malnutrition, undernutrition, nutritional risk, or related outcomes;
(3) Addressed the involvement, characteristics, burden, interventions, barriers and facilitators of informal caregivers in nutritional care;
(4) Were published in English to ensure accessibility and relevance to the target audience and to avoid potential translation errors.

**Figure 1:**
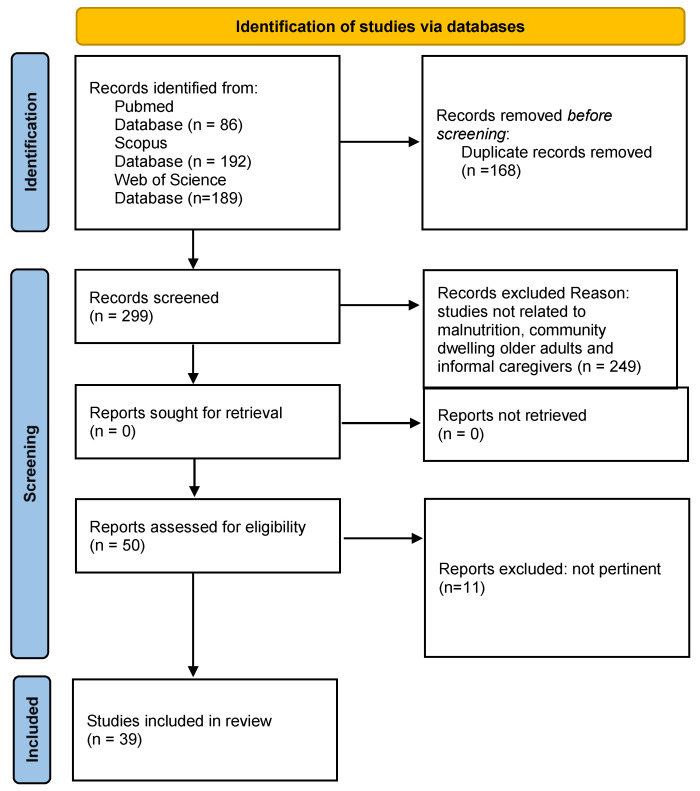
Flowchart to illustrate the selection and filtering process of articles in this narrative review (PRISMA model readjustment).

**Figure 2:**
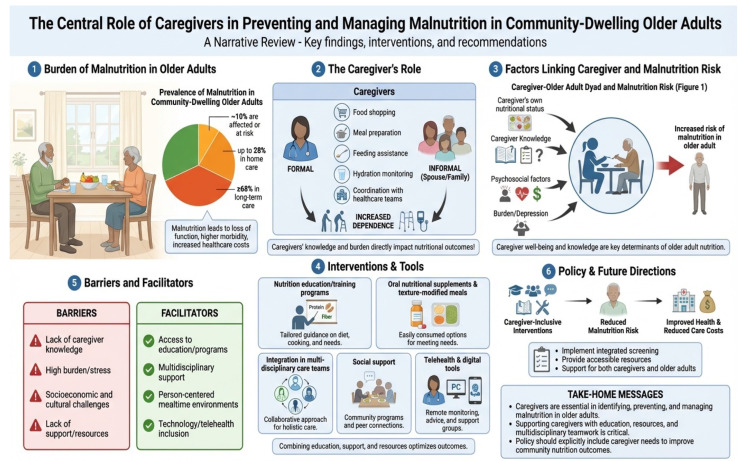
Conceptual framework demonstrating the role of the caregiver-related factors in the development of malnutrition among older adults.

Both primary studies and review articles were included to provide a comprehensive overview. Editorials, book chapters, study protocols, conference abstracts without full text and studies exclusively conducted in institutional settings were excluded. Primary studies were used to present detailed evidence, while review articles summarized broader trends and helped identify research gaps. Titles and abstracts were screened for relevance. Full texts of potentially eligible articles were assessed to confirm inclusion independently by two reviewers, with discrepancies resolved through discussion or consultation with a third reviewer. The co-authors discussed and agreed upon the final list of reference texts, totaling 34 (refer to Figure 1). Data were extracted and synthesized thematically. As this is a narrative review summarizing studies with heterogeneous designs (observational, qualitative, interventional, and meta-analyses), no formal quality assessment or standardized risk-of-bias tool was applied across all studies. Indeed, the primary aim was to provide a comprehensive overview of caregiver-related factors and interventions rather than to conduct a quantitative synthesis or meta-analysis. Nonetheless, methodological quality and study design were considered during interpretation of findings. Greater weight was given to systematic reviews and meta-analyses, longitudinal studies, and randomized controlled trials where available. Limitations of included studies were critically discussed in the synthesis. Given the narrative nature of this review, no formal systematic review protocol or meta-analysis was conducted. An AI-based tool (Figurelabs) was used to assist in the creation of Figure 2. No AI tools were used for data analysis or interpretation. All content was verified and approved by the authors.

## 3. Results

### 3.1. Malnutrition in Community-Dwelling Older Adults

Malnutrition, a condition deriving from an imbalance between nutrient intake and body requirements that results in altered body composition, reduced functional and cognitive functioning and adverse health outcomes [17], affects a considerable proportion of older adults, with variable prevalence depending on the explored setting, the geographical area and the applied screening/diagnostic tool [2,18]. Using the Mini Nutritional Assessment, the most widely employed instrument for identifying older adults with/at risk for malnutrition, the reported prevalence in the community ranges from 3.1% [19] to 5.8% [20], rising to 8.7% in those receiving home-care services and to 28.7% in long-term care facilities [19]. When also including those at risk of malnutrition, a condition in which individuals do not yet meet diagnostic criteria for malnutrition but have identifiable factors that significantly increase the likelihood of developing it, these estimates increase to 26.5% for older adults in domiciliary care [21] and to 68.8% for those in residential care homes [22]. A study conducted in Greece focusing on community-dwelling older adults receiving home care found that 28.1% were at risk of malnutrition and 20% were frankly malnourished according to the MNA [23]. The latter finding is in line with the results of Zugul et al. [24], who reported a prevalence of undernutrition of 15.8% in older adults receiving formal home care using SNAQ65+. Applying the GLIM criteria for the diagnosis of malnutrition [25], which requires at least one phenotypic and one etiologic criterion, the prevalence of malnutrition in community-dwelling older adults is even higher, in the range of 14.6–23.9% [26,27]. The observed variation in malnutrition prevalence among community-dwelling older adults using different screening/diagnostic tools can be attributed to differences in the conceptual frameworks, cutoff thresholds, and component criteria embedded within these instruments, which capture distinct dimensions of malnutrition and nutritional risk and therefore identify partially overlapping but non-identical populations.

Malnutrition is often associated with sarcopenia [28], co-occurring particularly in those with low BMI [29]. Diagnosing sarcopenia in those who are malnourished is important because this condition is associated with an additional risk of functional disability, comorbidities and cognitive impairment [30]. The malnutrition–sarcopenia syndrome is clinically characterized by the combined presence of reduced nutrient intake, inflammation, loss of body weight, decreased muscle mass and strength, and impaired physical function, all of which collectively contribute to poorer clinical and functional outcomes [31]. According to the ESPEN guidelines on definitions and terminology in clinical nutrition sarcopenia, malnutrition/undernutrition and micronutrient deficiencies are included among the nutrition disorders and nutrition-related conditions, which also comprise overweight, obesity and refeeding syndrome [4]. While older adults are at risk of most of these disorders, undernutrition in the form of protein–energy malnutrition (PEM) is likely the most common form of malnutrition in community-dwelling seniors [5,6]. Poor appetite, the need for support with food-related issues and oral health problems are among the most represented contributing factors to undernutrition in this group of individuals [32]. Malnutrition resulting from poor nutritional intake in the community setting has been associated with falls, frailty, functional decline, rehospitalizations and increased morbidity and mortality [5]. In addition, low energy and protein intake can result in micronutrient deficiencies because of the reduced consumption of nutrient dense foods. On the other hand, micronutrient deficiencies, defined as insufficient intake, absorption, or utilization of one or more essential vitamins or minerals, leading to impaired physiological function and, in some cases, clinical symptoms, are associated with a range of health issues, including frailty, cognitive decline and reduced quality of life [6]. A recent study examining nutritional intakes and nutritional status in institutionalized and non-institutionalized older adults showed that malnutrition due to poor energy intake was associated with inadequate intakes of key macro- and micronutrients including proteins, fiber, folates, vitamins B6, Na, K, calcium, magnesium and vitamin B12 [33]. These findings are consistent with those of Vural et al., who showed reduced intakes of zinc, selenium, iron, copper and iodine in both settings [34].

Several factors contribute to malnutrition in community-dwelling older adults, including poor oral health, swallowing disorders, age-related anorexia and changes affecting gastrointestinal nutrient absorption, acute and chronic diseases and medications. Also, depression and other mental conditions, as well as socioeconomic and environmental issues contributing to food insecurity, play an important role [7,35]. Stratidaki et al. [23], investigating factors contributing to the nutritional status of older adults receiving home care, found that nutritional risk assessed by the MNA was significantly associated with oral hygiene, comorbidities, level of independence and mental status. In individuals with/at risk for malnutrition, reversing the risk and correcting the condition is challenging because older adults are notably less likely to change established eating behaviors and habits than younger individuals [36]. In addition, within the community, living conditions and care needs influence the prevalence/risk of malnutrition, which typically grows as dependency and support requirement increase. In a survey conducted among nutritionally dependent older adults, Van Wymelbeke-Delannoy et al. [37] found that culinary dependence was associated with overt malnutrition or increased risk measured by the MNA. The relative prevalence was 4% in those who were independent, 12% in older adults needing help with activities other than food assistance and 41% in those needing support for food-related activities. In the latter group a further distinction was made considering the type of support needed. Malnutrition risk was 46% in those who delegated food activities to a caregiver, rising to 60% and 69% in those using meals on wheels and living in long-term care, respectively [37]. Thus, reliance on caregivers for daily food-related activities can be considered an additional risk factor for malnutrition in this vulnerable population. Indeed, food dependency implicates not only the need for assistance with meal consumption, but also with food choice, provision and preparation. The consequences include the inability to choose plates according to personal preferences and dietary needs with potential adverse outcomes linked to the risk of weight loss, frailty or worsening of preexisting comorbidities. In addition, caregiver’s nutrition literacy, cultural background and preferences influence food choices and the recipient’s diet.

Malnutrition in the community setting has severe consequences, impacting the development of geriatric syndromes including frailty, fatigue and sarcopenia, higher risk of falls and osteoporotic fractures, micronutrient deficiencies and weakened immune function, impaired wound healing, and higher morbidity and mortality rates [18,38]. In addition, the use of healthcare resources (hospitalizations and readmissions, increased length of hospital stays, complications, loss of independence) is significantly increased by malnutrition [22], emphasizing the relevance of prevention programs from an economic perspective. In the community setting, early warning signs of malnutrition including unintentional weight loss, poor appetite or reduced food intake, increased falls or fractures, fatigue, lethargy, or confusion and social isolation or depression should therefore be recognized by caregivers and periodically monitored.

### 3.2. Caregivers in Older Adult Nutrition

Both formal (namely professional home care workers, nurses, social and occupational therapists) and informal (including nonprofessional home care workers, spouses, relatives and family members) caregivers are involved in the nutritional assistance of community-dwelling older adults. With regard to formal caregivers, several factors influence the choice of the caregiver, including care recipients’ levels of needs, general independence and available resources. Indeed, although formal caregivers may be assigned to the care of patients with highly complex care needs and low levels of independence but provide assistance on a time-limited and structured basis, informal caregivers are continuously engaged in caregiving activities and often provide ongoing support without defined time boundaries. Nutritional assistance may involve several tasks, including not only food provision, meal planning and cooking, feeding support and preparation of texture-modified diets but also preserving oral health and hydration, monitoring dietary intakes and weight changes and coordinating interventions with other healthcare providers. This significant involvement generates a relevant physical and emotional burden. Understanding which caregiver-related factors contribute to malnutrition enables the recognition of key areas for intervention.

### 3.3. Caregiver Attributes Related to Malnutrition

Poor nutritional status of community-dwelling older adults has a significant impact on caregiver burden, even higher than disability [39]. On the other hand, several caregiver-related factors influence recipients’ nutritional status and diet quality.

#### 3.3.1. Caregiver Nutritional Status

A few observational studies demonstrate a link between the caregiver’s and care recipient’s nutritional status, particularly in the setting of dementia. Tombini et al. reported a prevalence of malnutrition among caregivers of older adults with Alzheimer’s disease of 23.3% [40], while Rullier et al. observed a prevalence of 5.4 [41]. In the latter study, the nutritional status of the care recipient, assessed by the MNA, was highly correlated with that of the family caregiver [41]. Poor nutrient intake and limited food variety have been described both in older adults’ caregivers as well as in care recipients [42]. Hossain et al. [43] observed an inverse association between caregiving for an older adult and diet quality, while Koponen et al. found that inadequate energy intake is common in older family caregivers and is associated with a high burden of comorbidities [44]. Other studies showed that also factors like caregiving intensity and duration, older age, insomnia, lower education, and poor functional and cognitive status are associated with worse nutritional status in caregivers [39,45,46]. In addition, another observational study reported that, independently of MNA scores, >50% of caregivers show inadequate intakes of proteins, vitamin A, folate and fiber, while up to 40% do not meet the recommended dietary intakes for other important vitamins and micronutrients, including vitamin D and E, thiamine, magnesium and selenium [47]. Adequate protein intake is essential for older adults to preserve muscle mass, strength and mobility and to support overall health and independence [48], while adequate fiber intake supports gastrointestinal, metabolic and cardiovascular health [49]. Current guidelines suggest a protein intake of 1–1.2 g/kg/day for healthy older adults and up to 1.5–2 g/kg/day for those with acute or chronic conditions, as well as a daily intake of 25 g of fiber [50]. Conversely, a good nutritional status in the caregiver is positively associated with better a MNA score in the older care recipient [44]. Thus, for a caregiver, it is essential to understand the importance of a healthy diet to support their own care and their recipient’s health, to prevent malnutrition and to improve overall quality of life.

#### 3.3.2. Caregiver’s Knowledge and Nutrition Literacy

In community-dwelling older adults, the multifactorial causes of malnutrition involving physical, cognitive, and social issues pose several challenges to the diagnosis and effective management of the condition. Without specific education and training, picking up on subtle signs and broad manifestations of malnutrition, including muscle wasting, impaired wound healing and reduced immune function, may be difficult. Indeed, the level of nutritional knowledge among caregivers of older adults varies, but studies suggest significant gaps. One study found that the education level of the caregiver was significantly associated with the nutritional status of the care recipient, while income was not [51]. Similarly, a lack of awareness and knowledge about malnutrition and proper management during the transition phase from hospital to home care has been reported by Ten Cate et al. [52]. Two surveys conducted among caregivers of older community-dwelling adults with disability [53] and dementia [54] with both nutritional dependence and manifest nutritional issues and feeding problems also demonstrated limited knowledge and competences in nutritional management. Interestingly, another case–control study suggested that caregivers recognize overt signs of malnutrition (namely low BMI, unintentional weight loss) and respond by increasing nutritional provision; however, they may lack knowledge or tools for early detection of increased nutritional risk, overlooking warning signs, including anorexia, functional changes and social withdrawal until malnutrition occurs [55]. It should also be noted that a rapid increase in energy provision in undernourished individuals may increase the risk of refeeding syndrome, a potentially serious complication that can be overlooked.

Other key deficits in nutrition knowledge include understanding specific issues related to nutrition in older adults, including energy needs and nutrients for age-related conditions and diet- and disease-related issues during the aging process [53]. Education level and prior nutrition training are strong predictors of better knowledge and practices, highlighting the need for targeted educational programs; such programs should foresee the inclusion of nutritionists within multidisciplinary teams to ensure specialized guidance and comprehensive nutritional management

#### 3.3.3. Psychosocial, Environmental and Socioeconomic Factors

Several aging-associated factors have been associated with dietary changes and preferences in older adults. Physiological modifications involving hormonal profile, energy expenditure, absorption and metabolism of nutrients as well as impaired appetite, taste and smell contribute to reduced energy intake [56]. Other factors including psychosocial and cultural influences, economic status, autonomy and food insecurity are also involved [1,7,35]. According to a previous systematic review, factors like living in family, having a good educational level and income were the most important determinants of healthy dietary choices and eating behaviors among older community-dwelling adults [57]. Indeed, those who live at home with a caregiver and rely on caregiver’s assistance for nutrition-associated issues may display several needs related to food preferences and comorbidities. Thus, caregivers’ knowledge of culturally appropriate food options tailored to care recipients’ diverse backgrounds is highly relevant. In addition, the provision of nutritionally appropriate meals that account for underlying medical conditions—such as texture-modified diets for individuals with dysphagia and dietary adaptations for patients with chronic renal failure, gastrointestinal diseases, or diabetes—is essential. Psychosocial aspects of the dining environment, including mealtime assistance in a friendly and supportive environment, with visually appealing meal presentation, are notably associated with improved intake among older adults and should be prioritized [58]. Loneliness and depression are deeply associated with anorexia and are major contributors to malnutrition [57]. In contrast, family/shared mealtime can counteract age-related appetite loss and reduced food intake and improve energy intake in older adults [59].

Socioeconomic factors also play an important role in malnutrition in the community. Food insecurity involving both older adults and their family caregivers has been reported, with a variable prevalence according to geographical areas [60,61]. A previous study conducted in Finland showed that among a group of older females caring for their partners, 20% were at risk of malnutrition and 30% experienced subjective poverty affecting their ability to access and consume healthy foods [61]. These findings were confirmed by another study performed in the United States in a cohort of 594 caregivers assisting mostly older adults, which showed that caregiving was associated with greater risk of food insecurity (OR = 2.10) and personal hunger (OR = 2.89) [62]. These findings highlight the need of targeted education and training programs among community health professionals focusing on the social determinants of malnutrition and on possible interventions.

#### 3.3.4. Caregiver Burden and Depression

Independently of nutritional status, caregivers of dependent community-dwelling older adults often experience substantial burden, largely driven by psychological stress, physical strain, and the time devoted to caregiving tasks [63]. A systematic review aimed at identifying the consequences of the burnout syndrome in informal caregivers of older patients with dementia found that it negatively affects caregivers’ quality of life and generates anxiety and depression in the care recipient [64]. Poor nutritional status may further exacerbate this burden by increasing care complexity and placing additional demands on caregivers. Tana et al., investigating the association of older care recipients’ nutritional status and caregiver burden found that malnutrition in the care partner was independently more associated with the caregiver’s burden and stress than cognitive and physical disability [39]. Indeed, the increasing burden on family caregivers of older adults with chronic conditions, particularly with dementia, is tightly associated with poor nutritional and functional status in the care recipient [65,66]. A cross-sectional study investigating factors associated with poor caregiver nutritional status in the caregiver–older care recipients with dementia dyad found that caregiver depression measured with the CES-D score was associated with malnutrition, assessed by the MNA, demonstrating that caregiver depression compromises caregiver nutritional status [67] and can affect the quality of nutritional care. Indeed, a study focusing on a group of family caregivers examined the factors that hindered the success of a nutritional intervention based on nutritional guidance. The findings indicated that depressive symptoms were associated with poorer improvements in nutritional status, with caregivers reporting fewer depressive symptoms being more likely to achieve positive changes in Mini Nutritional Assessment scores during the study period [68]. On the other hand, higher self-compassion in caregivers of older adults is associated with better nutritional status in care recipients [69].

A conceptual framework linking caregiver-related factors and malnutrition in older care recipients is shown in Figure 2, and a summary of caregiver involvement and malnutrition outcomes in care recipients is provided in Table 1.

**Table 1:** Observational studies examining caregiver involvement and malnutrition outcomes in older adults.

Author (Year)	Country	Study Design	Population and Setting	Caregiver Type	Nutrition-Related Outcomes	Key Findings
Tana C (2019) [39]	Italy	Cross-sectional	Community-dwelling older adults	Informal (family)	Caregiver Burden Inventory	Caregiver burden linked to impaired ADL and nutritional status
Rullier L (2013) [41]	France	Cross-sectional	Community-dwelling older adults with dementia	Informal (family)	MNA (caregiver and care recipient), MMSE, ADL and NPI (care recipient)	Caregiver’s nutritional status is associated with that of care recipient’s
Hossain S (2021) [43]	US	Cross-sectional	Community-dwelling older adults	Informal (family)	Healthy Eating Index-2010	Caring for elderly is inversely linked to caregiver’s diet quality
Koponen S (2024) [44]	Finland	Cross-sectional	Community-dwelling older adults	Informal (family)	MNA	Better recipient MNA scores are associated with higher handgrip and better caregiver MNA scores
Savela R-M (2023) [61]	Finland	Cross-sectional	Community-dwelling older adults with dementia	Informal (family)	Caregiver’s MNA and food insecurity	20% of female caregivers are at risk of malnutrition and 30% experience poverty, dietary inadequacy and higher burden of comorbidities
Rullier L (2014) [67]	France	Cross-sectional	Community-dwelling older adults	Informal (family)	MNA (caregiver and care recipient), depression (caregiver), apathy, and dependency (care recipient)	Worse caregiver nutritional status is associated with caregiver depression and with higher care recipient dependency and poorer nutritional status
Bozkir C (2025) [69]	Germany	Cross-sectional	Community-dwelling older adults with dementia	Informal (family)	MNA, depression (care recipient), and caregiver self-compassion	Higher self-compassion in caregivers is associated with improved nutritional status in elderly care recipients

MMSE: Mini Mental Status Examination; ADL: activities of daily living; MNA: Mini Nutritional Assessment; NPI: Neuropsychiatric Inventory.

### 3.4. Nutritional Interventions Involving Caregivers

Despite limited knowledge and other factors that impact care recipients’ nutritional status, caregivers of disabled older adults demonstrate proactive practices towards nutritional management. A recent cross-sectional study investigated the knowledge, attitudes, and practices of caregivers of functionally disabled older adults regarding nutritional management. The findings revealed that caregivers generally reported proactive nutritional practices, such as planning meals, monitoring hydration, and adapting food texture to swallowing abilities. However, notable knowledge gaps persisted, particularly in more specialized aspects of nutritional care, including micronutrient requirements, monitoring weight changes, and managing enteral feeding procedures [53].

Indeed, a seminal systematic review assessed the effectiveness of nutrition-related interventions delivered to or by informal caregivers and non-clinical community care workers for community-dwelling older adults. Nine intervention studies of moderate quality were included, most of which involved informal caregivers and aimed at identifying, preventing, and/or treating malnutrition. Interventions targeted at malnutrition were generally able to improve or stabilize the nutritional status of older adults assessed by MNA, MST or PG-SGA and, in some cases, prevent further decline in functional status. Importantly, these nutrition interventions did not increase the burden of informal caregivers. Overall, these findings support the inclusion of informal caregivers as part of the nutritional care team for older adults living in the community [70]. In a follow up narrative review, the same authors identified several nutrition-related tasks in which family caregivers of older adults are involved and concluded that the evidence for the efficacy of caregivers’ delivered interventions aimed at implementing nutrition screening and referral pathways is moderate, as is the evidence supporting the integration of family caregivers into nutritional care teams [71]. Altogether, these findings support the importance of nutrition interventions targeting older adults’ caregivers to optimize caregiving behaviors and burden and, ultimately, the nutritional status and nutrition-related outcomes of care recipients. In addition, due to the multifactorial causes of malnutrition, attitudinal barriers and caregiver burden should be prioritized in a holistic approach that integrates caregivers’ and older adults’ needs [72]. Based on caregiver involvement, three types of interventions can be identified. New technologies, foreseeing different types of caregiver involvement depending on the technology used, will be addressed separately.

#### 3.4.1. Caregiver-Focused Interventions

Several interventions are designed with the primary aim to support and educate the caregiver in order to improve knowledge, skills and confidence in the care recipient’s nutrition management and to reduce the burden of caregiving [73]. Nutrition and education programs and tools and caregiver stress reduction programs and support groups are included in this category and are supported by several studies. Educational programs generally aim at enhancing caregivers’ understanding of malnutrition, its association with prevalent geriatric conditions such as Alzheimer’s disease and Parkinson’s disease, and practical strategies to optimize daily nutrient intake and clinical outcomes [74]. For example, a randomized controlled trial assessing the effects of educational sessions delivered to home caregivers of dependent older adults by nurses demonstrated that the intervention improved nutritional status assessed by the MNA in the recipients [75]. In addition, a systematic review by Rea et al. [76] showed that nutrition education devoted to caregivers of older adults reduces the risk of malnutrition assessed by the MNA and the probability of hospital readmission following discharge from the hospital. Education delivery strategies include group education, skill development workshops, distribution of educational material and telehealth. Importantly, two randomized clinical studies showed that both educational sessions and nutritional counseling can improve nutritional status assessed by MNA, body weight, macro- and micronutrient intake and quality of life in the dyads [77,78]. Two other studies conducted among informal caregivers of older adults with cognitive impairment confirmed that nutrition education and counseling conducted by telephone or by online courses had a positive effect on nutritional knowledge and status, improved abnormal eating behaviors and decreased caregiver’s burden [79,80]. Educational interventions should not be limited to the prevention/treatment of malnutrition. Indeed, potential underlying causes, including oropharyngeal issues (poor dentition, dysphagia and presbyphagia), health-related conditions (chronic diseases, cognitive impairment and polypharmacy), social and psychological factors (loneliness and depression) and environmental/economic hurdles, should be identified and addressed. Also, when delivering an educational intervention, contents should be clear and appropriately adapted to the recipients’ level of knowledge and literacy. To this regard, a study conducted among informal caregivers of community-dwelling older adults with dementia found that the provided nutrition-related material was unclear and unsuitable for their level of knowledge. In addition, more nutritional advice specific to the disease and less referrals were advocated [81]. Another study conducted among caregivers of cognitively impaired older adults, using a mixed-method approach, showed poor knowledge about disease-specific food choices according to the Mediterranean–DASH Intervention for Neurodegenerative Delay (MIND) diet recommendations and high burden of stress and exhaustion, calling for specific dietician-driven education [82]. Programs should emphasize not only the technical knowledge of nutrition but also the importance of caregiver support, engagement, empowerment and training.

Regarding older adults at nutritional risk, another relevant field is that of training programs focused on the preparation of high-protein–energy-dense meals and food fortification. Indeed, evidence shows that protein intake falls below recommended values in older adults [83] and that factors including aging-associated anorexia [84] and low awareness of the importance of proteins in aging contribute to reduced intake [85]. Modified-texture meals are a necessity for older adults with chewing and swallowing difficulties, such as those affected by dysphagia or by neurological disorders. However, inadequate caregiver know-how on texture modification can result in meals that are nutritionally inadequate, unappealing or unsafe, thereby increasing the risk of undernutrition and aspiration while significantly impacting caregiver burden and stress [86]. Educational programs targeting caregivers may improve knowledge of texture classifications, proper food preparation techniques, nutritional adequacy and palatability, representing key interventions to support safe feeding practices and optimize nutritional status.

A summary of nutritional outcomes of education interventions targeting informal caregivers of older adults via different tools is reported in Table 2. The table includes studies published since 2020; previous evidence has been exhaustively summarized by Rea et al. [76] and by Marshall et al. [70,71].

#### 3.4.2. Caregiver-Inclusive Interventions

A few interventions target the older adult but actively include the caregiver with the goal of improving nutritional outcomes by involving the dyad in planning and implementation. These include, for example, dietitian and nutritionist consultations, home-based nutrition assessment with caregiver participation, behavioral change programs, disease-specific nutrition management and post-hospital discharge nutrition planning.

In a randomized control trial, Suominen et al. assessed the effects of tailored nutritional guidance delivered by nutritionists to caregivers of older adults with dementia over a one-year period. Nutritional guidance improved protein intake and quality of life in the intervention group [87]. A systematic review considering variable formats of interventions, including family caregivers of community-dwelling older adults, assessed the impact of delivered interventions on nutritional and other health-related outcomes [70]. Results support the role of caregivers in conducting nutritional interventions when assisted by professionals, in particular dietitians. Integration of caregivers into nutrition care teams and collaboration with other health professionals including nurses, primary care providers and social workers is also effective in preventing and treating malnutrition, particularly in patients with dementia.

#### 3.4.3. Caregiver-Delivered Interventions

The third type of interventions includes those where the caregiver actively delivers the nutrition support to the older adult with the goal of directly implementing nutritional care strategies in the domiciliary setting. Examples of this type of intervention include providing oral nutritional supplements and home enteral nutrition, preparing texture-modified or fortified meals and monitoring nutritional and hydration status.

In older adults, when adequate nutritional intakes cannot be achieved through oral nutrition or protein-fortified foods, oral nutritional supplements (ONSs) should be used [50]. ONSs are classified as foods for specific medicinal purposes, typically dispensed through pharmacies and used under the supervision of qualified health professionals, such as physicians and dieticians and nutritionists. This strategy has been adopted in patients discharged from hospitals to long-term care, with several studies conducted among nursing homes residents and very few among older adults living at home with a caregiver [88]. A systematic review and meta-analysis assessing the effects of ONS intervention in frail community-dwelling older adults found a slight positive effect on energy and protein intake, mobility and quality of life; analysis of cost-effectiveness was inconclusive due to poor evidence (only one economic evaluation available) [89]. Another meta-analysis conducted among community-dwelling older adults without severe comorbidities showed beneficial effects of ONSs on anthropometric and functional parameters [90]. Importantly data on adherence to ONS prescription are sparse due to the poor methodological quality of interventional studies [91]. Adherence rates reported in the literature typically range from 48 to 100% of prescribed intake, with substantial variability [92]. Even modest reductions in intake (≥20%) can attenuate the expected benefits [92]. This is relevant since caregivers play an important role in facilitating and monitoring ONS acceptance and consumption.

With regard to dysphagia, the availability of ready-made texture-modified and fortified meals appears to be promising both from a nutritional and safety perspective, as it greatly reduces the risk of aspiration—common in post-stroke patients—while alleviating caregiver burden [93].

When oral nutrition is unsafe or insufficient, artificial nutrition such as enteral or parenteral feeding is provided, and enteral nutrition, in particular, requires significant involvement from caregivers to manage feeding tubes, formulas, and daily nutrition administration. In the study by Silver et al. [94], family caregivers of older adults discharged on home enteral nutrition reported performing many complex care tasks and expressed substantial training needs, especially for technical and nutrition-related aspects of enteral feeding. Caregiver preparedness scores were generally low, but higher preparedness was associated with greater perceived competence and effectiveness and lower health care use. The authors concluded that caregivers need better multidisciplinary training and support—including dietitian involvement—to improve both caregiver effectiveness and enteral nutrition outcomes for community-dwelling older adults.

#### 3.4.4. Technology-Based and Innovative Approaches

A growing body of evidences shows promising effects of telehealth, digital nutrition tools and remote monitoring and reminder systems in supporting community-dwelling older adults and their caregivers [95].

Information and communication technologies for the management of nutrition in older adults include home-based and mobile device sensors, smart devices, fitness devices, assistive robots, tablets, computers and smartphones, and videoconferencing resources [96] and support several nutrition-related caregiver tasks. Mobile health applications enable caregivers to track dietary intake, set reminders for meals and supplements, and access evidence-based nutrition guidance in real time. These apps can support individualized meal planning, help monitor fluid intake, and facilitate communication with healthcare providers when needed. Wearable devices and sensors offer another promising opportunity by enabling passive monitoring of several parameters and activities including eating behaviors. When paired with diet tracking tools, such technologies can help caregivers identify patterns of inadequate intake, dehydration risk, or changes in functional status that may signal emerging malnutrition. Telehealth and remote consultations enable dietitians and nutrition specialists to connect with older adults and their caregivers in home settings. This facilitates tailored nutritional assessments, education, and real-time feedback without the need for in-person visits. The opportunities offered by technology-led interventions and their effects have been assessed by a few studies and reviews.

One systematic review and meta-analysis targeting seniors living in the community and assessing the effect of interventions delivered via telephone and internet-enabled telemedicine devices found increased protein intake and improved quality of life with a trend towards better nutritional status, physical function and reduced hospital readmissions and mortality [97]. Despite these findings, several challenges remain. Many existing tools lack robust validation in older adult populations and fail to integrate into caregivers’ daily routines. Technology adoption can be hindered by usability issues, digital literacy gaps, privacy concerns, and limited interoperability with healthcare systems [98] highlighting the need for dedicated training and ongoing technical support [99]. Informal caregivers may also experience increased cognitive and emotional load when managing multiple platforms or interpreting complex health data. Therefore, despite the considerable potential offered by new technologies for the management of nutritional care of community-dwelling older adults by their informal caregiver, their full exploitation still appears limited.

## 4. Discussion

Effective management of malnutrition in community-dwelling older adults requires a multifaceted approach that emphasizes improving caregivers’ nutritional knowledge, integrating caregiver support into nutrition care models, including caregivers in nutritional screening, and developing targeted strategies that enhance care quality while reducing caregiver burden.

### 4.1. Barriers and Facilitators to Implementing Caregiver’s Driven Nutritional Interventions: Implications for Policies and Practices

Nutrition-related issues regarding older adults and their caregivers still represent an unmet need. A small descriptive qualitative study assessing needs of informal caregivers to support older adults’ healthy diets recognized several barriers, involving poor education and communication in the dyads, negative attitudes in caregivers, environmental restrictions and limited resources. In contrast, social support, access to healthy foods, proper nutrition knowledge, positive attitudes and sufficient resources were acknowledged as facilitators [100]. Another study assessing nutritional care in the transition from hospital to home care identified several gaps, including a general lack of a comprehensive approach to nutritional care in the context of aging and disease, the absence of individualized nutritional care at home, mutual incomprehension and missed shared decision making, as well as lack of support to the caregiver [101]. A scoping review including 24 studies aiming at assessing the roles of informal caregivers of older community-dwelling older adults identified several challenges and barriers related to system fragmentation and a lack of interface coordination roles [102]. Launholt et al., in their scoping review, identified additional barriers to the implementation of nutrition-targeted interventions for community-dwelling older adults and healthcare professionals/caregivers, including a lack of knowledge and awareness, resources and collaboration/communication, missing links between the different healthcare settings and poor insight among older adults and their caregivers. In contrast, education and training, self-care, person-centered care, technology in nutrition care, and social and psychological factors were identified as potential facilitators [103].

These cues offer a valuable opportunity to develop nutritional strategies policies and interventions targeting older adults and their family caregivers to tackle malnutrition.

#### 4.1.1. Improving Caregiver’s Nutritional Knowledge

Caregiver-focused nutrition knowledge assessment tools and guidance are a prerequisite for improving nutritional knowledge and dietary habits [104]. Caregiver-oriented guidelines and recommendations addressing topics such as determinants of malnutrition in older adults, malnutrition risk screening and assessment, food fortification and texture modifications, and ONSs are available and should be implemented [105,106,107], involving dietitians and nutrition health professionals for guidance and supervision [108].

#### 4.1.2. Integration of Caregiver Support into Nutrition Care Models

Since caregivers have a direct influence on the nutritional status of older adults, it is important to include informal caregivers in the identification, prevention, and management of malnutrition in order to mitigate its consequences. The development of targeted nutritional interventions that also involve caregivers under the supervision of nutritional health professionals may contribute to reducing the risk of malnutrition [71].

#### 4.1.3. Importance of Caregiver-Inclusive Nutritional Screening

Some studies indicate that the risk of malnutrition in older adults is higher in care dyads where the caregiver is malnourished. This association may be explained by similar eating behaviors and dietary habits developed during cohabitation. Given the prevalence of malnutrition and the associated risks, regular assessment and improvement of the nutritional status of older adult–caregiver dyads should be integrated into healthcare practice.

#### 4.1.4. Recommendations for Caregiver-Focused Nutrition Strategies and Caregiver Burden

It is necessary to adopt corrective measures aimed at the early identification of nutritional deficiencies and malnutrition risk in both patients and their caregivers, while improving the intake of essential nutrients. Some studies suggest gender differences in the ability to cope with nutrition-related caregiving responsibilities, with older adults cared for by male caregivers being at higher risk of malnutrition [109]. Traditional gender roles may limit cooking skills and confidence, contributing to poorer dietary outcomes for both caregivers and care recipients. Caregivers with limited resources may also experience difficulties in providing nutritious meals, increasing the risk of unhealthy eating habits for themselves and those they care for. Since loneliness negatively affects eating behaviors, organizing shared meals with family members may represent a strategy to increase caloric intake among homebound older adults.

### 4.2. Implications for Long-Term Care and Home Care Services

Several studies highlight a desire for greater nutritional knowledge among both caregivers and patients, alongside an acknowledged lack of adequate information [72,73]. In this regard, educational interventions that also integrate behavioral change strategies may be useful in promoting the adoption and maintenance of healthier dietary habits. Such education should consider the specific nutritional requirements of older age, such as generally higher protein needs. Interventions should also promote practical skills related to identifying, evaluating, and applying nutritional information. In addition, practical structural interventions should be implemented to support healthy eating among community-dwelling older adults and their informal caregivers.

### 4.3. Relevance for Aging-in-Place Models and Long-Term Care Policy

Malnutrition places a substantial burden on health, social, and long-term care systems in terms of costs related to hospital admissions and care provided in nursing homes and home care services. In light of this evidence, interdisciplinary collaboration within home care systems, including the involvement of qualified dietitians, appears necessary to provide personalized guidance and support older adults and caregivers in implementing dietary recommendations in everyday life. The implementation of long-term nutritional interventions also including oral nutritional supplements and medically tailored meals is essential to prevent early malnutrition risk and to reduce treatment-related costs [110].

### 4.4. Limitations, Gaps in the Literature and Future Research Directions

This narrative review has several limitations, including substantial heterogeneity among the included studies in terms of design, population characteristics, interventions, and outcome measures, which may limit the comparability of findings and the generalizability of the conclusions. Many studies are based on pre- and post-intervention designs, non-randomized, or involve small sample sizes, which limits the strength of evidence and the ability to draw causal conclusions. In addition, the geographical concentration of studies in certain regions and the limited inclusion of culturally diverse populations may affect the generalizability of the findings to broader community-based settings. Finally, some studies may not fully represent the heterogeneity of community-dwelling older adults, particularly regarding functional status, socioeconomic factors, and caregiving contexts. As a narrative review, this study is inherently subject to potential selection and interpretation bias, as the identification, appraisal, and synthesis of evidence were not conducted using a systematic search strategy or standardized risk-of-bias assessment framework. In addition, restriction to articles published in English may have led to the exclusion of inherent studies available in other languages and introduced a language bias. Nonetheless, significant gaps emerge from the literature regarding the nutritional status of dependent community-dwelling older adults and their caregivers. In general, caregivers, particularly informal, represent an understudied population as well as their older care recipients. The lack of high-quality longitudinal and intervention studies, as well as standardized outcome measures related to caregiver involvement, limits the reliability and utility of their results for guiding effective interventions. Several factors may contribute to this gap, including difficulties in recruiting large populations and high drop-out rates related to the characteristics of older adult populations. Factors such as regional differences, cultural norms related to food and caregiving, and access to nutritional resources may influence the relevance of study findings. Also, insufficient data are available about the economic impact of interventions addressing malnutrition among community-dwelling older adults, particularly in relation to the costs borne by informal caregivers and the inclusion of diverse cultural contexts that may shape the access to support services. There is a clear need for further intervention studies addressing the complexity of malnutrition etiology and management in real-world settings. Future research should aim to demonstrate the long-term impact of targeted nutritional interventions on malnutrition risk. In addition, few interventions consider the cultural, social and environmental contexts of caregivers living in the community. Finally, there is little research on effective educational tools or support systems that assist caregivers in the provision of proper nutrition for older adults. These shortfalls indicate areas for possible improvement and opportunities for precision and personalized nutrition approaches. Future interventions should include evidence-based caregiver training programs, including in-presence and digital resources, and implementation of community support networks to reduce caregiver stress and share resources. Culturally sensitive and individualized approaches, including interventions adapted to family routines, local food availability and cultural preference, should be implemented to improve adherence and outcomes. Finally, future research and development should focus on new technologies, particularly on the integration of artificial intelligence to provide personalized nutrition recommendations based on real-time data while minimizing caregiver burden. By leveraging technology thoughtfully and collaboratively, future interventions have the potential to empower informal caregivers, enhance the nutritional care of older adults, and ultimately improve health outcomes in this vulnerable population.

## 5. Conclusions

Protein–energy malnutrition affects a relevant number of community-dwelling older adults who are dependent on their caregivers for several nutrition-related issues. Despite the substantial impact of informal caregivers on shaping the nutritional status of care recipients, there remains a marked lack of high-quality literature, robust study designs, and well-structured interventional models to effectively guide practice and policy in this area. Thus, the results of this review support a call for integrated caregiver-focused nutrition strategies, including caregiver-inclusive nutrition research and policy development.

## Figures and Tables

**Table 2:** Informal caregiver-focused or caregiver-inclusive nutritional interventions in older community-dwelling adults from studies published since 2020.

Author (Year)	Country	Study Design	Population and Setting	Caregiver Role	Intervention Description	Nutrition-Related Outcomes	Main Findings
Koponen S (2022) [77]	Finland	RCT	Community-dwelling older adults	Informal (family)	Nutritional counseling versus standard of care	Dietary intake	Individualized nutritional counseling improves protein, vitamin D, and calcium intake
Meriç ÇS (2025) [78]	Turkey	Pre–post intervention	Dependent community-dwelling older adults	Formal and informal	Nutritional education sessions conducted by nutritionists	MNA at 0 and 12 months, QoL, dietary intake, anthropometry, and functional and biochemical parameters	Significant improvements in MNA, QoL, body weight outcomes and in intakes of energy, proteins, carbohydrates, vitamin B12, C, calcium and phosphorus
Hsiao H-T (2020) [79]	Taiwan	Non-randomized clinical trial	Community-dwelling older adults with dementia	Informal (family)	Routine care and telephone counseling versus structured DDEP	MNA at 0, 3 and 6 months; Caregiver’s Healthy Eating Behavior for Dementia Questionnaire	Improved caregiver nutritional knowledge and healthy eating in the DDEP group; no significant changes in MNA scores
Lin YJ (2024) [80]	Taiwan	Quasi-experimental	Community-dwelling older adults with dementia	Informal	Nutritional education via online course	Abnormal eating behavior, nutritional status, and caregiver burden at 0, 1 and 3 months	Intervention improved abnormal eating behavior and nutritional status and decreased caregiver burden

MNA: Mini Nutritional assessment; QoL; quality of life: DDEP: dementia dietary program.

## Data Availability

No new data were created or analyzed in this study. Data sharing is not applicable to this article.

## Supplementary Materials

The following supporting information can be downloaded at: https://www.mdpi.com/article/10.3390/nu18060982/s1.

## Abbreviations

The following abbreviations are used in this manuscript:

MNA	Mini Nutritional Assessment
SNAQ65+	Short Nutritional Assessment Questionnaire 65+
BMI	Body Mass Index
ESPEN	European Society for Enteral and Parenteral Nutrition
Na	Sodium
K	Potassium
MMSE	Mini Mental Status Examination
ADL	Activities of Daily Living
NPI	Neuropsychiatric Inventory
ONS	Oral Nutritional Supplement
QoL	Quality of life
DDEP	Dementia Dietary Education Program
