# Supplementary material for: The Role of Caregivers in Preventing and Managing Malnutrition Among Older Adults: A Narrative Review
Source: Nutrients. 2026 Mar 19;18(6):982. doi: 10.3390/nu18060982 (PMC13029402; doi:10.3390/nu18060982)
Supplement: Supplementary file 1 [file nutrients-18-00982-s001.zip › nutrients-4162491-supplementary.pdf]

Supplementary Table S1

Database	String for literature search	Retrieved records (n)
Pubmed	("Malnutrition"[Mesh] OR malnutrition[tiab] OR undernutrition[tiab] OR "Protein-Energy Malnutrition"[Mesh] OR "nutritional status"[tiab]) AND ("Aged"[Mesh] OR "Aged, 80 and over"[Mesh] OR older adult*[tiab] OR elderly[tiab] OR geriatric*[tiab]) AND ("Caregivers"[Mesh] OR caregiver*[tiab] OR "informal caregiver*"[tiab] OR "family caregiver*"[tiab] OR "spousal caregiver*"[tiab]) AND (depend*[tiab] OR "care-dependent"[tiab] OR "activities of daily living"[Mesh] OR ADL[tiab] OR disability[tiab]) AND (determinant*[tiab] OR predictor*[tiab] OR risk factor*[tiab] OR "caregiver burden"[Mesh] OR "caregiver burden"[tiab] OR "caregiver stress"[tiab] OR barrier*[tiab] OR facilitator*[tiab] OR ("Nutrition Therapy"[Mesh] OR intervention*[tiab] OR program*[tiab] OR education[tiab] OR training[tiab]))	82
Scopus	TITLE-ABS-KEY (malnutrition OR undernutrition OR "protein-energy malnutrition" OR "nutritional status" OR "nutrition risk")<br>AND TITLE-ABS-KEY ("older adult*" OR elderly OR geriatric* OR "aged 65" OR "aged 80 and over")<br>AND TITLE-ABS-KEY (caregiver* OR "informal caregiver*" OR "family caregiver*" OR "spousal caregiver*")<br>AND TITLE-ABS-KEY (depend* OR "care-dependent" OR "activities of daily living" OR ADL OR disability OR frail*)<br>AND TITLE-ABS-KEY (determinant* OR predictor* OR "risk factor*" OR "caregiver burden" OR "caregiver stress" OR barrier* OR facilitator* OR intervention* OR program* OR education OR training)	192
Web of Science (Topic search)	((malnutrition OR undernutrition OR "protein-energy malnutrition" OR "nutritional status" OR "nutrition risk") AND ("older adult*" OR elderly OR geriatric* OR "aged 65" OR "aged 80") AND (caregiver* OR "informal caregiver*" OR "family caregiver*" OR "spousal caregiver*") AND (depend* OR "care-dependent" OR "activities of daily living" OR ADL OR disability OR frail*) AND (determinant* OR predictor* OR "risk factor*" OR "caregiver burden" OR "caregiver stress" OR barrier* OR facilitator* OR intervention* OR program* OR education OR training))	189
